# Synthesis of an efficient MOF catalyst for the degradation of OPDs using TPA derived from PET waste bottles

**DOI:** 10.1038/s41598-023-46635-6

**Published:** 2023-11-06

**Authors:** Hossein Yarahmadi, Sultan K. Salamah, Marwan Kheimi

**Affiliations:** 1 Department of Chemical Engineering, Sirjan University of Technology, Sirjan, Iran; 2 Civil Engineering Department, College of Engineering, Taibah University, P.O. Box 30002, 41447 Al-Madina, Saudi Arabia; 3 Department of Civil and Environmental Engineering, Faculty of Engineering—Rabigh Branch, King Abdulaziz University, 21589 Jeddah, Saudi Arabia

**Keywords:** Heterogeneous catalysis, Pollution remediation, Organic-inorganic nanostructures

## Abstract

In this study, a method for degrading PET-waste plastic bottles using ZnCl_2_:Urea as a catalyst was developed, resulting in high conversion (87%). The terephthalic acid obtained from the degradation of Waste PET Bottles (WPTs) was combined with copper and zinc salts to synthesize bimetallic metal–organic frameworks (MOF). The effectiveness of a bimetallic Cu-Zn(BDC)-MOF in catalyzing the reduction reaction of organic pollutant dyes (OPDs) was investigated, and the degradation efficiency of individual dyes was optimized, achieving over 95% degradation within 6–12 min under optimal conditions. Various techniques, including FT-IR, XRD, FE-SEM, EDS, and TEM were used to characterize the synthesized MOF. Results showed that the catalytic activity of Cu-Zn-MOF in reduction reaction of OPDs was enhanced by increasing the copper content. The reaction kinetics were investigated following pseudo-first-order kinetics with rate constants of 0.581, 0.43, 0.37, and 0.30 min^−1^ for Methylene Blue (MB), Methyl Orange (MO), 4-Nitrophenol (4-NP), and 4-Nitroaniline (4-NA), respectively. The investigations revealed that the produced catalyst exhibited excellent stability and recoverability, while its activity remained well-preserved even after undergoing three reuse cycles.

## Introduction

Industrial wastewater in developing countries is often contaminated with textile organic dyes and heavy metals, leading to high concentrations of pollutants in water resources^1,2^. The widespread use of stable, hazardous dyes, such as Methyl Orange, Methylene Blue and 4-Nitro phenol, in various industries including textiles, food, plastics, cosmetics, and carpets has raised concerns among researchers^1,3^. These pollutants have been continuously released into the environment for decades, posing a threat to human health and the environment. They are resistant to biological treatment methods and can persist in surface, ground, and drinking water^4–6^. Contaminated drinking water can exhibit genotoxicity and mutagenicity; therefore, efficient water treatment methods are necessary to effectively remove and separate hazardous dyes from wastewater^7^.

Various natural and synthetic environmental remediation strategies have been reported for the treatment and remediation of contaminated wastewater. These include conventional techniques such as biochemical and biological processes^8^, membrane and filtration treatments^9^, adsorption^10^, coagulation-flocculation^11,12^, centrifugation^13^, chemical (or electrochemical) reduction/oxidation procedures, and catalytic degradation^14,15^. Catalytic degradation is a promising method and one of the most widely used procedures for environmental pollution treatment^15,16^.

An ideal catalyst for catalytic degradation should be stable under aqueous acid or alkaline conditions, have high porosity, be cost-effective, easy to apply and retrieve, and have other desirable properties^17–20^. The effectiveness of a catalytic method depends on factors such as selectivity, kinetics, fast mass transfer, strong host–guest interactions, high specific surface areas (SSA), low-cost preparation, flexible approach to their preparation, recyclability, reusability, etc.^21,22^. To fulfill these requirements, researchers have focused on developing and optimizing novel alternative low-cost catalysts with high catalytic efficiency and recyclability such as activated carbon, coal, clay, fly ash, zeolites, and metal–organic frameworks (MOFs)^23–25^.

MOFs are promising materials for various applications due to their advantageous characteristics, including high surface area, porosity, and stability^25^. They have been widely studied and applied in fields such as catalytic reactions, photosynthesis, and wastewater purification^23^. MOFs offer improved performance compared to traditional materials in terms of adsorption–desorption, removal, separation or degradation of pollutants such as heavy metals, CO_2_, CH_4_ and OPDs^25^. Their tunable structure and pore size, as well as the presence of metal sites, make them a versatile option for enhancing water quality and reducing environmental impact in industrial processes^26,27^. In the realm of literature, bimetallic MOFs have emerged as a novel development, displaying the potential for catalytic enhancement through mixed metal samples^28^. The dispersion of metallic components such as Fe, Zn, or Cu throughout MOFs may also serve as active sites in hetero-catalytic reduction reactions for pollutant organic contaminants. However, there remains room for improvement in the catalytic performance of these MOFs. The effectiveness of MOF-catalyzed reduction reactions is closely linked to their metallic components^29,30^. Incorporating active metal centers into pristine MOFs presents an effective means of increasing their catalytic activity^31^. Given the redox activity of copper, its doping as the second metal has been proposed as a way to enhance the catalytic performance of MOFs^32,33^. Specifically, partial substitution of zinc centers with Cu in Zn-based MOFs may serve to boost the catalytic efficiency of MOFs in the degradation of OPD^34,35^.

This study aims to examine the potential of bimetallic MOFs in enhancing catalytic activity for water treatment purposes. The focus is on the preparation of modified bimetallic nanocomposite Cu-Zn-MOFs using WPTS, commonly known as "white pollution." The aim is to investigate the catalytic ability of MOFs in the degradation of OPDs in aqueous media under mild conditions. A bimetallic Cu-Zn(BDC)-MOF was successfully synthesized and utilized as a heterogeneous catalyst in this work. This cost-effective and green approach involved using TPA on MOF structure produced from the degradation of WPTS, with the incorporation of Cu to explore deep catalytic efficiency for the chemical degradation of OPDs.

The synthesized MOF was structurally confirmed using various techniques such as Fourier-transform infrared spectroscopy (FT-IR), X-ray diffraction (XRD), field emission scanning electron microscope (FE-SEM), Transmission Electron Microscope (TEM) and Energy-dispersive X-ray spectroscopy (EDS) techniques. The study also investigated the effects of MOF dose, initial concentration of OPDs and NaBH4, kinetics, recyclability and reusability of bimetallic Cu-Zn(BDC)-MOF. This innovative advancement offers a resolution to the pressing issues associated with the contamination caused by ecologically dangerous OPDs.

## Material and methods

### Chemicals and materials

All chemicals (Zn(OAc)_2_·2H_2_O, Cu(NO_3_)_3_·2H_2_O, ZnCl_2_·H_2_O, Urea, Ethylene glycol (EG), Dimethyl formamide (DMF), Ethanol (EtOH), Methyl orange (MO), Methylene blue (MB), 4-Nitro phenol (4-NP) and 4-Nitro aniline (4-NA)) were prepared by Merck and Sigma-Aldrich Chemical. All chemicals were analytical grade and used without further refining. The used WPBs were purchased from public recycling center.

### Catalytic depolymerization of PET

The degradation of polyethylene bottles was conducted with minor modifications, using the method proposed in the literature^36^. PET water bottles were purchased from recycling facilities. Initially, 5 g of the waste plastic-bottle was washed, dried, and cut into 5 × 5 mm pieces. Afterward, the PET fragments were placed into a 50 mL glass flask with three necks, containing 20 g of EG and equipped with a reflux condenser and thermometer. Next, 0.25 g of the ZnCl_2_:Urea as the catalyst, with mole ratios of 1:6, was added to the mixture. The glycolysis of PET was carried out under atmospheric pressure for 30 min at 170 °C, utilizing a magnetic stirrer and an oil bath. Upon completion of the degradation reaction, the mixture was cooled to room temperature. Cold distilled water (1000 mL) was then added, the pH was adjusted to 3–4 using H_2_SO_4_ (2 M) to obtain a white slurry, and the resulting product was filtered to separate it from the residual PET pellets. The remaining PET was subsequently dried at 70 °C until it reached a constant weight, and the conversion of PET was calculated to be 87% using the following formula:

$$Conversion\,of\,PET ({\%})=(\frac{initial\,weight\,of\,PET - weight\,of\,residual\,PET}{initial\,weight\,of\,PET})\times 100$$

The degradation products were isolated and purified using the separation techniques outlined in literature sources^37,38^. Subsequently, the filtrate was concentrated through the use of a vacuum rotary evaporator at 80 °C and then placed in a refrigerator at 0 °C for a period of 5 h. Following this, white crystals of terephthalate acid (TPA) monomer precipitated.

### Preparation of MOF samples

Using a solvothermal method, Cu-Zn(TPA)-MOF bimetallic compounds were prepared in accordance with a previously established process^28,39^. The synthesis of all organic–inorganic frameworks investigated in this study was achieved by employing terephthalic acid, a compound derived from the degradation of polyethylene plastic bottles. The synthesis involved combining 5 mmol each of Zn(OAc)_2_·2H_2_O and Cu(NO_3_)_2_·3H_2_O in a 100 mL beaker with 5 mmol of TPA. A homogeneous solution was obtained by slowly adding 60 mL of DMF at room temperature, utilizing magnetic stirring for approximately 30 min. The resultant precursor solution was then placed into a Teflon-lined stainless steel autoclave and heated for 36 h at 110 °C. After cooling, the bright-blue precipitate was collected through centrifugation and washed with deionized water, ethanol, and DMF solvents. Then, the sample was dried in a vacuum oven at 80 °C for 12 h. This produced the bimetallic Cu-Zn-MOF. Additionally, two monometallic MOF samples (Zn-MOF and Cu-MOF) were created using the same method, but by adding only Zn(OAc)_2_·2H_2_O or Cu(NO_3_)_2_·3H_2_O, respectively, for comparison purposes.

### Degradation experiment

In general procedure, 20 mL of desired OPDs solution with an initial concentration of 20 ppm was combined with MOF (dosage: 1–4 mg) in individual beakers. Following the addition of the catalyst to the dye-containing solution, the resulting mixture is stirred for 10 min using a magnetic stirrer. Subsequently, the sodium borohydride solution is introduced (15–35 mL, 0.1 M). It is important to note that the moment of adding the sodium borohydride solution marks the initiation of the desired reaction. The suspension was stirred continuously with a magnetic stirrer at room temperature. At regular time intervals, 2.5 mL samples were withdrawn from the reaction solution, quenched with 5 mL distilled water, and the used catalyst was separated by centrifugation. The dye degradation proceeding was quantified by UV–Vis spectroscopy for the residual dye by measuring the absorption changes at the corresponding maximum wavelength.

## Result and discussion

### Characterization of the synthesized TPA and MOFs

The crystal structure of the prepared MOFs was identified using the D8-ADVANCE XRD gauge (Bruker, Germany) with 10 to 90° Cu-Ka radiation at a scan rate of 2° per minute. The surface morphology and Energy-dispersive X-ray spectroscopy analysis (EDS) of the synthesized samples were recorded by a TESCAN MIRA III Field Emission Scanning Electron Microscopy (FE-SEM) at a voltage of 15.0 kV. FT-IR was used to characterize the solid MOFs and their spectra were recorded in the range of 400–4000 (KBr, cm^−1^) wavenumbers on a JASCO 6300 spectrophotometer. Additionally, Transmission electron microscopy (TEM) images were obtained using Philips EM 208S. The BDC monomer formed from PET depolymerization was characterized by ^1^H-NMR spectra assigned on a Bruker (Avance DRX-400 MHz) spectrometer relative to tetramethylsilane (TMS = 0.00 ppm) as an internal standard in DMSO-*d*_*6*_. The progression of the OPD reduction reactions was evaluated using a UV–Vis spectrophotometer equipped with a quartz cell, and absorbance measurements were recorded.

#### ^1^H-NMR analysis of prepared TPA

**Figure 1:**
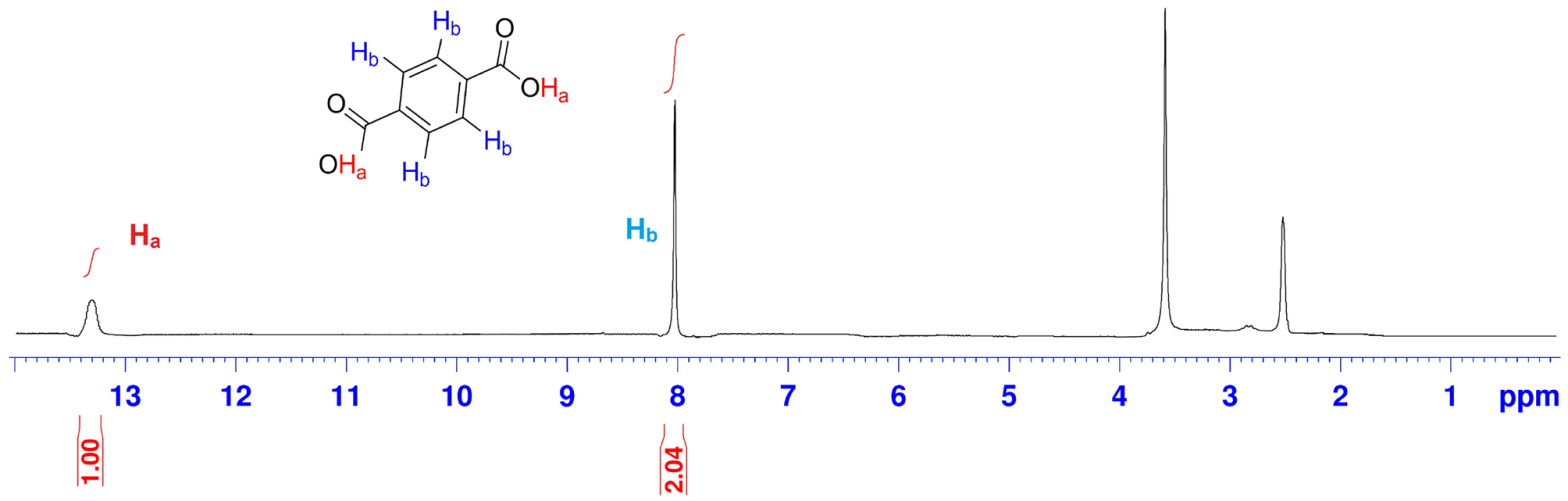
^1^H-NMR of the prepared TPA.

The ^1^H-NMR technique was utilized to confirm the production of TPA. ^1^H-NMR (400 MHz, DMSO-*d*_*6*_): δ = 13.28 (–OH) and 8.05 (aromatic C–H) ppm (Fig. 1)^40^.

#### FT-IR analysis of prepared MOF

**Figure 2:**
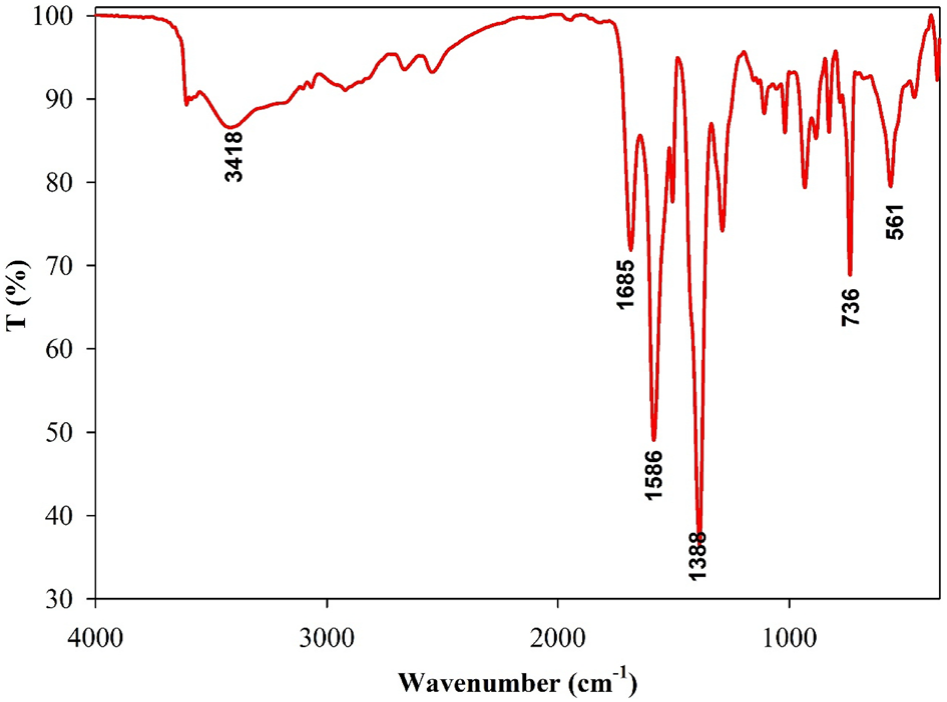
FT-IR of the synthesized Cu-Zn-MOF.

After synthesis, FT-IR spectroscopy was employed to characterize the structure of MOFs (Fig. 2). The FT-IR pattern and absorptions were observed to be almost identical to those of its organic precursor due to the same functional groups present in both MOF and TPA. By comparing the prominent observed peaks, it is evident that there is a satisfactory alignment with the findings reported in previous studies^41,42^. The broad stretching vibrational peak centered at 3418 cm^−1^ originated from the –OH in acidic groups, which may be attributed to free water adsorbed on the MOF surface or non-bonded carboxylate functional groups of TPA precursors in MOF structure. The characteristic absorption band at 1685 cm^−1^ was identified as the vibration of (O–C=O) carboxylate. The band at 1289 cm^−1^ was related to the vibration of C–O in TPA precursor. The stretching bands at 1685 and 1110 cm^−1^ were of high intensity and could be attributed to C=O and C–O bonds, respectively, present in the structure of TPA-Zn-Cu, indicating that these functional groups generate stable coordination interactions with metallic ions. A slight displacement of the carbonyl group-related absorption band was observed in MOFs compared to that in TPA due to its coordination bond with the Zn (or Cu) ion, resulting in a shift to lower frequencies from 1710 to 1685 cm^−1^. Additionally, the low-intensity vibrations related to the C=O and –OH bonds (compared to TPA) indicated stable coordination interactions of these functional groups with metallic ions in MOF structure. The assigned absorption band at 540 and 561 cm^−1^ in the resulting bimetallic MOF curve was related to the bending vibration of Zn–O and Cu–O linker modes, respectively. Peaks located at 1108 and 1018 cm^−1^ were related to 1,4-disubstitution of the benzene ring individually, and the bands at 1388 and 1586 cm^−1^ were assigned to C=C of aromatic benzene rings. The absorption peak at 736 cm^−1^ was attributed to the C–H out-of-plane bending vibration of unsaturated carbons in aromatic rings, while bands at 3200–3000 cm^−1^ were related to the stretching vibration of C–H bonds in benzene rings. The in-plane bending vibrations and the stretching vibrations of C=C of the benzene ring at 900 ~ 675 cm^−1^ were accompanied by absorption bands from 1289 to 1586 cm^−1^.

In this study, metal–organic frameworks (MOFs) were synthesized using organic linkers with a uniform structure, known as TPA. This uniformity in the linkers resulted in the formation of identical functional groups within the MOFs. Considering the similarity of these functional groups across the investigated MOF structures, it is reasonable to expect that there would be no significant differences in the FT-IR analysis peaks of these compounds.

#### X-ray diffraction

**Figure 3:**
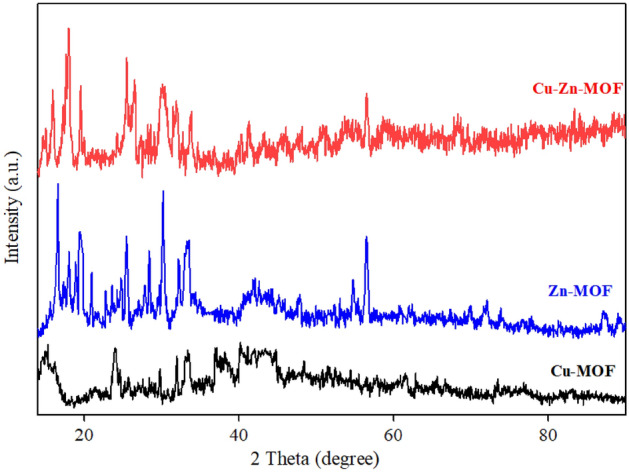
XRD of MOFs (Cu-MOF, Zn-MOF and Cu-Zn-MOF).

The utilization of XRD is a highly effective approach to investigating the crystalline properties of synthesized materials. In this context, the XRD patterns for Cu-MOF, Zn-MOF and bimetallic Cu-Zn-MOF are presented in Fig. 3. It is noteworthy that Bragg diffraction results in high intensity peaks at 2θ = 12.2°, 14.4°, 15.0°, 15.4°, 17.8°, 19.3°, 24.0°, 25.1°, 26.3°, 28.4°, 30.1°, 33.8° and 56.4° which confirm successful synthesis of the Cu-MOF, Zn-MOF and bimetallic Cu-Zn-MOF. By comparing the prominent peaks observed, such as peaks 17.8°, 26.3°, 25.1°, 33.8° and 56.4° in the XRD patterns, it is evident that there is a satisfactory alignment with the findings reported in previous studies^41–44^. The comparison of the synthesized MOF graphs demonstrates that altering the metal ratios within the respective MOF structures has minimal impact on the overall pattern. The observed peaks exhibit a high degree of reproducibility, albeit with slight deviations. Notably, the peaks in the Cu-MOF graph appear to be relatively weaker, which could be attributed to the lower crystallinity exhibited by this sample. This finding suggests that the Cu-MOF structure possesses a higher degree of amorphousness. Furthermore, increasing the zinc-to-copper ratio in the composition enhances the intensity of the peaks, indicating a significant improvement in the crystallization of structures containing zinc. These results shed light on the relationship between metal ratios and crystallinity in MOF structures. The presence of specific impurity peaks in Cu-Zn-MOF can be attributed to the interactions that arise due to the bimetallic nature of the MOF structure. This observation highlights the significance of understanding the effects of bimetallic interactions in MOFs. The results obtained from XRD analysis demonstrate a significant resemblance between single-metal and bimetallic organic–inorganic framework structures. This finding can be attributed to the concept of "heterogeneous network chemistry," which suggests that the introduction of different metals into structural units containing multiple metals results in non-periodic changes in spatial arrangement while preserving the overall framework order of MOFs. These findings contribute to our understanding of the chemistry and design principles behind MOF structures, with implications for various fields including chemistry and environmental science.

#### SEM (Cu-Zn-MOFs)

**Figure 4:**
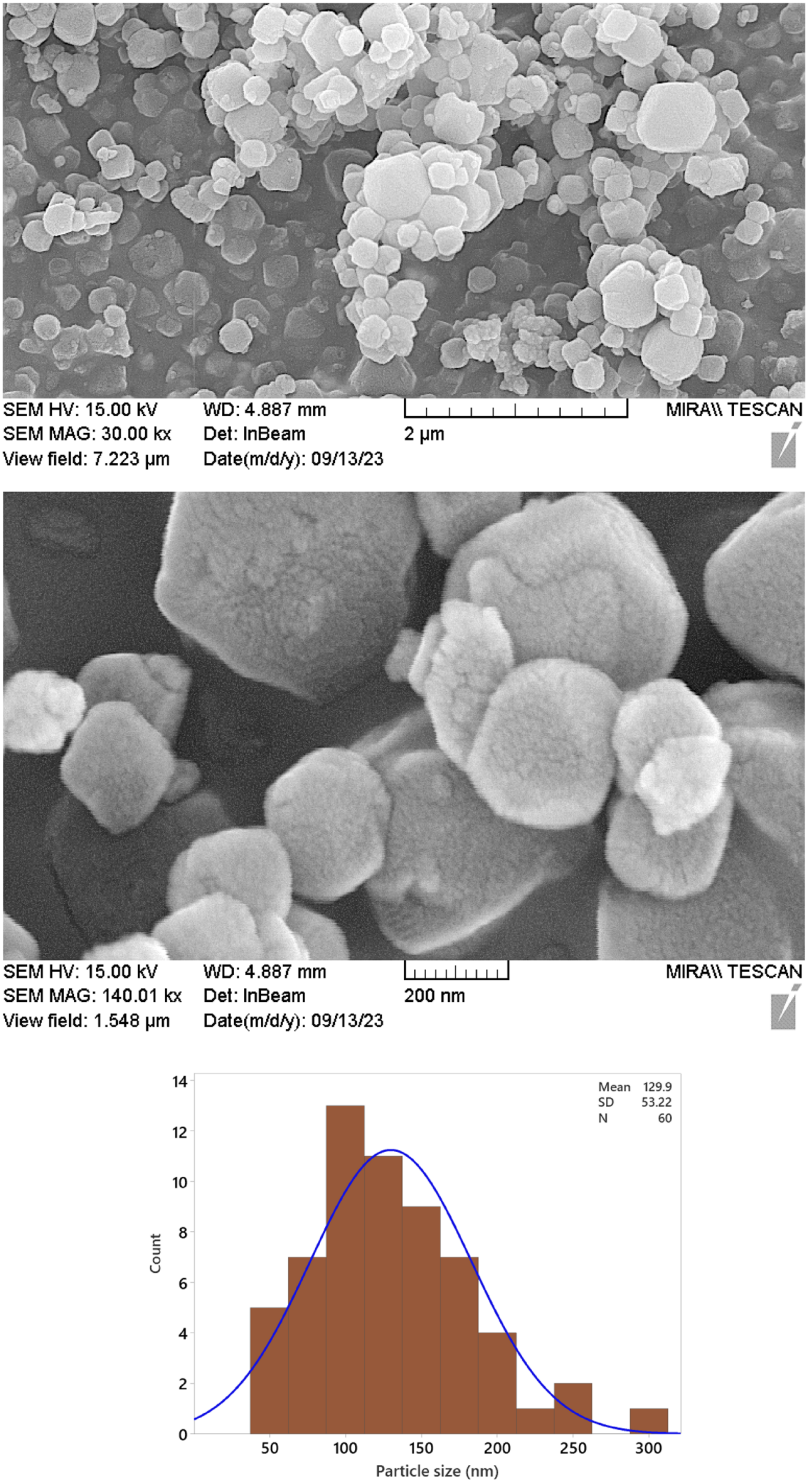
FE-SEM images and corresponding histogram of particle size distribution for Cu-Zn-MOFs.

The morphology and particle sizes of Cu-Zn-MOFs synthesized using degraded WPT were studied using FE-SEM (Fig. 4). The results show the distinct crystal structure exhibited by metal–organic frameworks (MOFs) with remarkable geometrical morphology. A comprehensive analysis of the synthesized MOF structures revealed a remarkable distribution of dimensions with an average particle size of approximately 130 ± 3 nm. This is consistent with previous literature^28^. The particle size histogram is shown in Fig. 4.

#### EDS analysis

**Figure 5:**
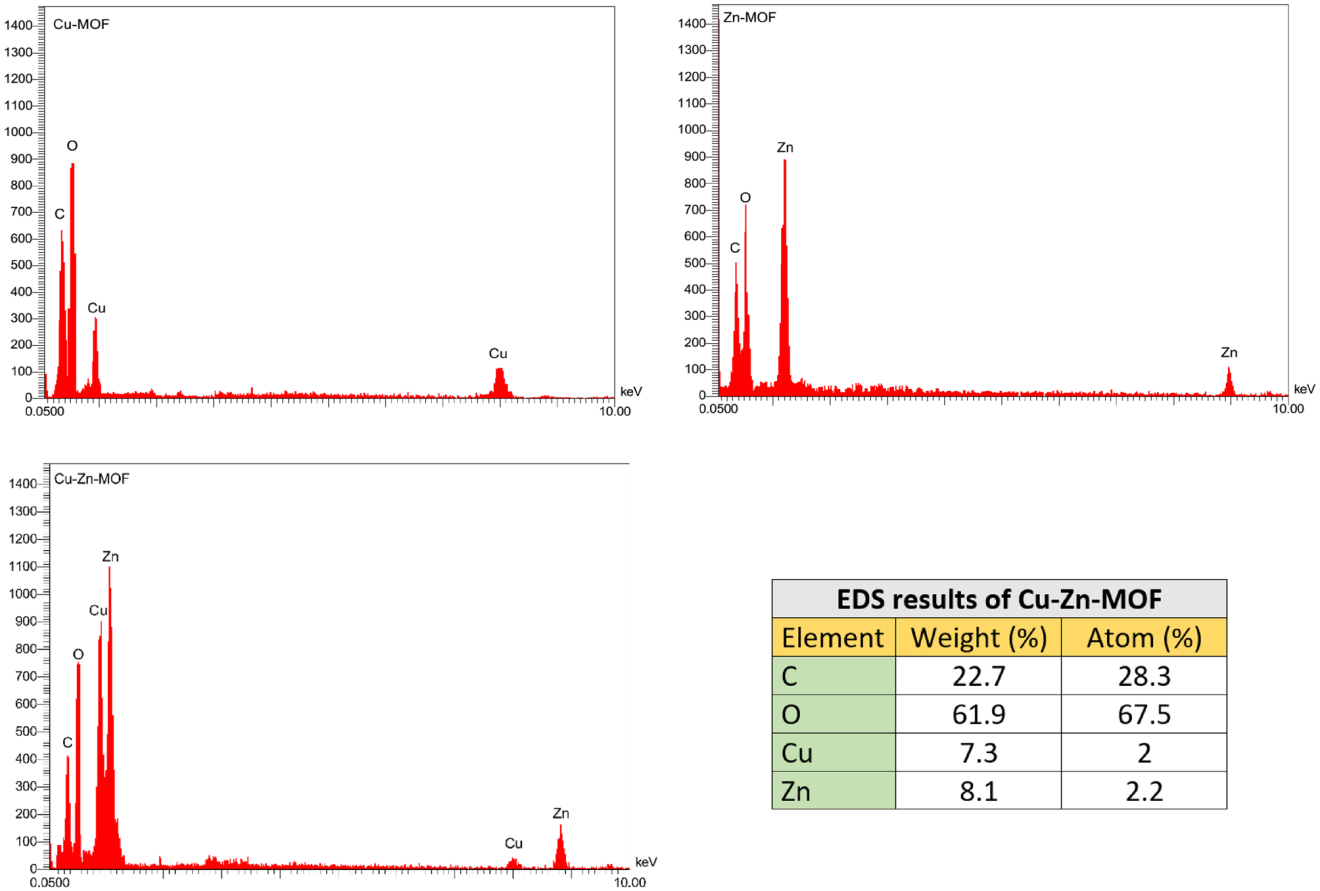
EDS analysis of synthesized MOFs (Cu-MOF, Zn-MOF and Cu-Zn-MOF).

The chemical composition of the synthesized Cu-Zn-MOFs was examined using energy dispersive spectroscopy (EDS) and the results confirmed the presence of copper, zinc, carbon, and oxygen (Fig. 5).

The table inserted in the EDS pattern displays the quantitative analysis of the constituent elements in Cu-Zn-MOF. The EDS analysis also revealed that the Cu-Zn-MOF consisted of 22.7% weight of carbon, 61.9% weight of oxygen, 7.3% weight of copper, and 8.1% weight of zinc. Furthermore, the atomic percentages of elements were determined as follows: carbon (31.6%), oxygen (64.5%), copper (1.9%), and zinc (2.0%).

#### TEM (Cu-Zn-MOFs)

**Figure 6:**
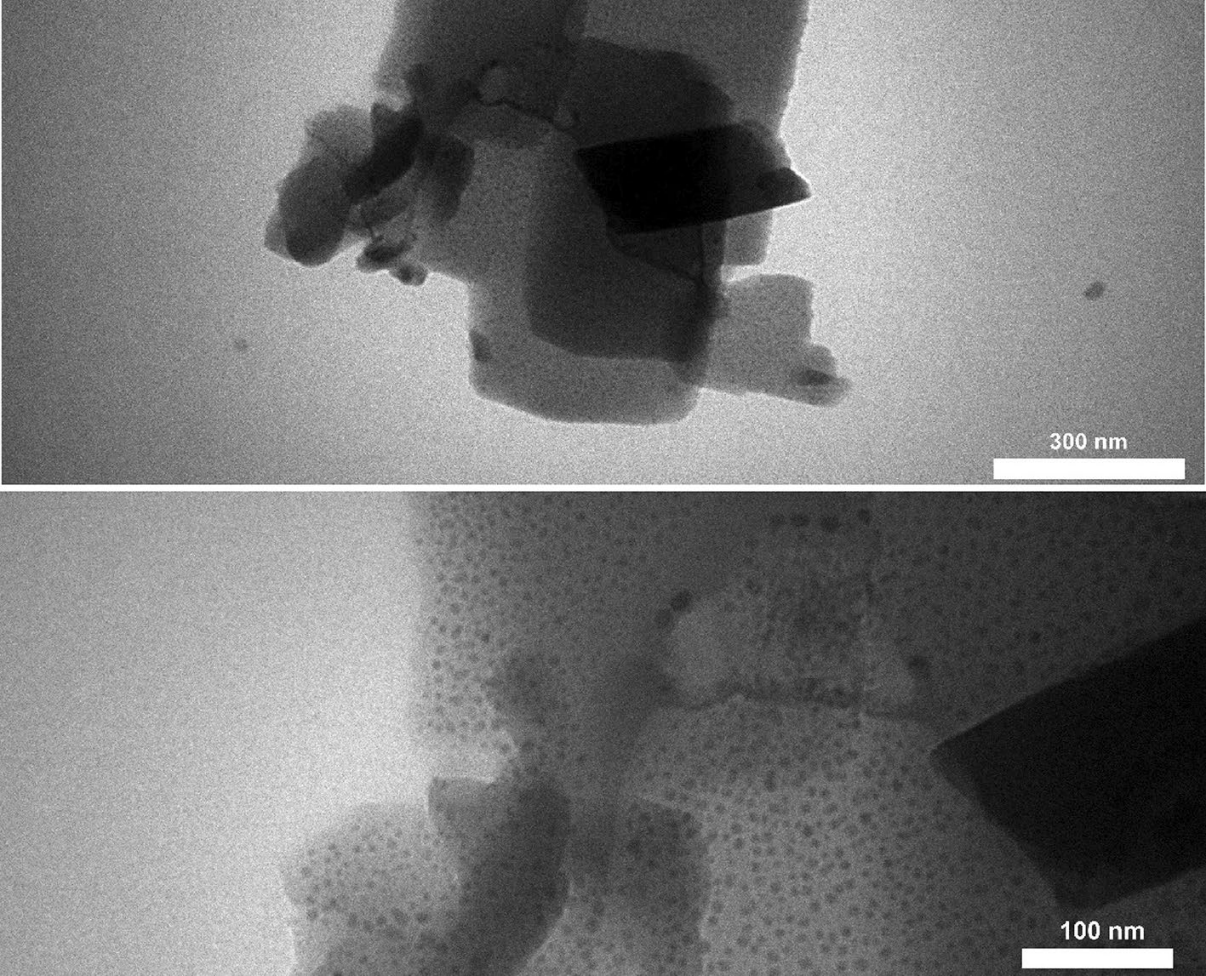
TEM of Cu-Zn-MOFs.

The morphology of Cu-Zn-MOFs was further studied using transmission electron microscopy (TEM), which showed that the MOFs had geometrical and cubic shapes with minimal aggregated structures (Fig. 6). The scattered dark black shadows observed on the pale gray background corresponded to the metal centers of Cu and Zn due to their greater electron density compared to other elements (C and O)^45^. The diameter of MOFs with nano-scale dimensions is clearly illustrated in Fig. 6, indicating an approximate overall diameter of 120 nm. The particle size and shape observed in TEM were consistent with FE-SEM.

#### Catalytic activity and Kinetic degradation reactions

To determine the dye-reduction ability of the synthesized MOFs, a batch technique was used to test individual dyes. Various factors including reaction time, efficiency, recoverability, and reusability of the catalyst were investigated to obtain optimal conditions for catalytic degradation of OPDs. The results were listed in Table 1.

**Table 1:** The used corresponding maximum wavelengths and volumes (or dosage) of reagents in catalytic room temperature degradation of OPDs in the presence of MOFs and NaBH_4_ (15–35 mL).

Entry	Dye/mL/ppm	λ_max_ (nm)	Catalyst (1–4 mg)	Time (min)	Degradation (%)
1	MB/20/20	663	Zn-MOF	60	< 15
2	MB/20/20	663	Cu-MOF	60	95–98
3	MO/20/20	465	Zn-MOF	60	< 10
4	MO/20/20	465	Cu-MOF	60	93–98
5	4-NP/20/20	400 (317)	Zn-MOF	60	< 10
6	4-NP/20/20	400 (317)	Cu-MOF	60	94–99
7	4-NA/20/20	380	Zn-MOF	60	< 8
8	4-NA/20/20	380	Cu-MOF	60	90–98
9	MB/20/20	663	Cu-Zn-MOF	5–10	93–99
10	MO/20/20	465	Cu-Zn-MOF	6–12	92–98
11	4-NP/20/20	400 (317)	Cu-Zn-MOF	6–12	90–98
12	4-NA/20/20	380	Cu-Zn-MOF	8–15	88–97

Based on the results, it can be inferred that the inclusion of copper into the metal–organic framework structure results in an enhancement of both the catalytic efficiency and the dye degradation (Table 1). It is also worth mentioning that MOF structures with a higher percentage of zinc, exhibited enhanced resistance to water washing, leading to significantly improved recyclability and reusability when compared to other structures under identical experimental conditions.

The degradation of dyes in the presence (or absence) of Cu-Zn-MOF and NaBH_4_ was investigated. To begin, a solution of the OPD (20 mL, 20 ppm) was prepared. Subsequently, 4 mg of the Cu-Zn-MOF catalyst was added to the solution, which was then stirred for 1 h at room temperature. Surprisingly, no significant catalytic degradation of the OPD was observed under these experimental conditions. Next, the degradation of the OPD was examined in the presence of sodium borohydride without the catalyst. For this purpose, a solution containing the target OPD (20 mL, 20 ppm) was prepared and then, sodium borohydride solution (30 mL, 0.1 M) was added to the mixture, which was stirred for 1 h. The degradation of various dyes under these conditions resulted in progress ranging from 6 to 11%. Notably, it is remarkable that the degradation and reduction of these dyes, in the absence of either catalyst or NaBH4, requires an extremely long time (more than 1 h) and yields very low efficiency. These findings emphasize the necessity of utilizing both NaBH_4_ and catalyst simultaneously in the OPD degradation, providing compelling evidence to support this approach.

**Figure 7:**
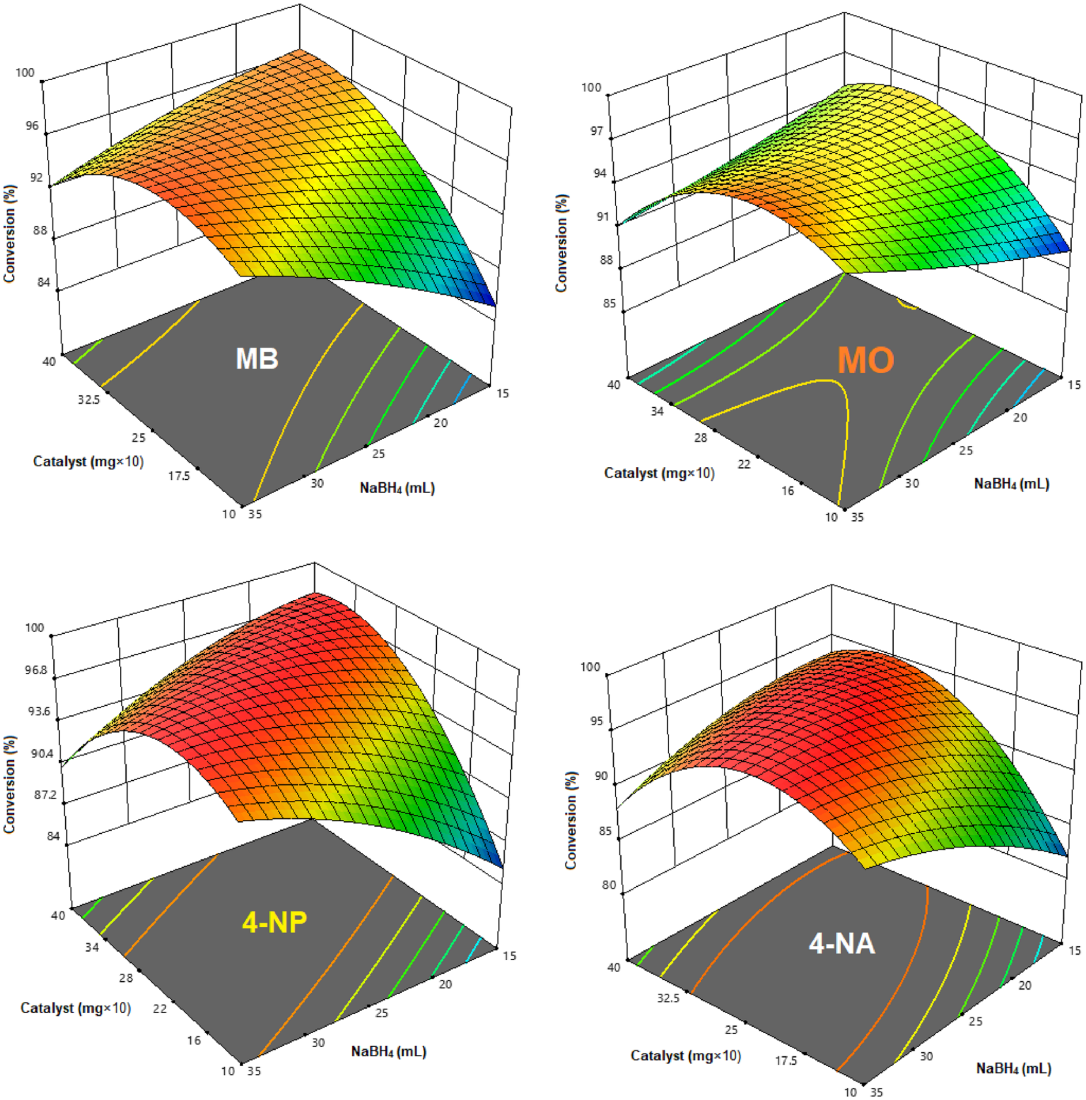
Optimizing of conditions for OPD degradation in the presence of different amounts of NaBH_4_ (0.1 M) and Cu-Zn-MOF.

After demonstrating the necessity of both MOF and NaBH_4_ for OPDs reduction, the catalytic efficiency of the prepared MOF catalyst was evaluated. The degradation of OPDs (MB, MO, 4-NP, and 4-NA) was monitored using UV–visible spectrophotometry in the presence of both MOF and NaBH_4_ as the typical reaction model. The catalytic performance of the prepared Cu-Zn-MOF samples was evaluated for the reduction of OPDs. Initially, to obtain the optimal conditions for degradation of the investigated OPDs in this study, based on the conditions listed in Table 1, the degradation reaction was executed for each OPD, separately. The obtained results were represented in Fig. 7.

Our experiments have demonstrated the quantitative degradation of OPDs using Cu-Zn-MOFs. The results showed that the presence of metal sites as redox-active centers in MOF structures was responsible for their efficient catalytic activity and improved catalytic performance. The addition of Cu-Zn-MOFs catalyst to the reaction medium resulted in a decrease in the intensity of the peak at 663, 465, 400, and 380 nm corresponding to MB, MO, 4-NP, and 4-NA, respectively^46–49^. We hypothesized that the Cu-centers on the high specific surface area of MOF could be well-suited for the reduction reaction of OPDs molecules, thereby enabling their efficiency in an aqueous mixture. The catalytic activity of MOF is expected to improve as the quantity of Cu increases in the degradation reaction. However, our results have demonstrated that the recyclability and reusability of MOF improves when more zinc metal is used in the structure of MOF (Table 1). Therefore, by considering two important factors of a catalyst (activity, recyclability and reusability), we prepared MOF with copper and zinc atoms in equal molar ratios and attempted to use them for the catalytic OPD degradation. The final optimal conditions for investigating the kinetic degradation reaction of OPDs can be observed in Table 2.

**Table 2:** The optimized conditions for the degradation of OPDs in the presence of Cu-Zn-MOF.

OPD/mL/ppm	Catalyst (mg)	NaBH_4_ (mL)	λ_max_ (nm)
MB/20/20	2.3	28	663
MO/20/20	3.0	25	465
4-NP/20/20	2.5	22	400 (315)
4-NA/20/20	3.0	20	380

Also, standard curves were used to measure the OPD concentrations at various points during the reaction (Fig. 7). It was observed that the bimetallic Cu-Zn-MOF can catalyze the reduction reaction of OPDs by more than 95% within 6–12 min under optimized conditions. Table 2 summarizes the values used to find optimal conditions, and the percentage degradation efficiency of MOFs was determined using the following equation where [A_o_] is the initial absorbance (λmax at 663, 465, 400 and 380 nm) and [A_t_] is the absorbance at the reaction time t in the catalytic reaction. [A_o_] is the initial concentration and [A_t_] is the concentration of OPDs in the catalytic experiment.

$$Degradation (\%)=\left(\frac{[Ao] - [At]}{[Ao]}\right)\times 100$$

**Figure 8:**
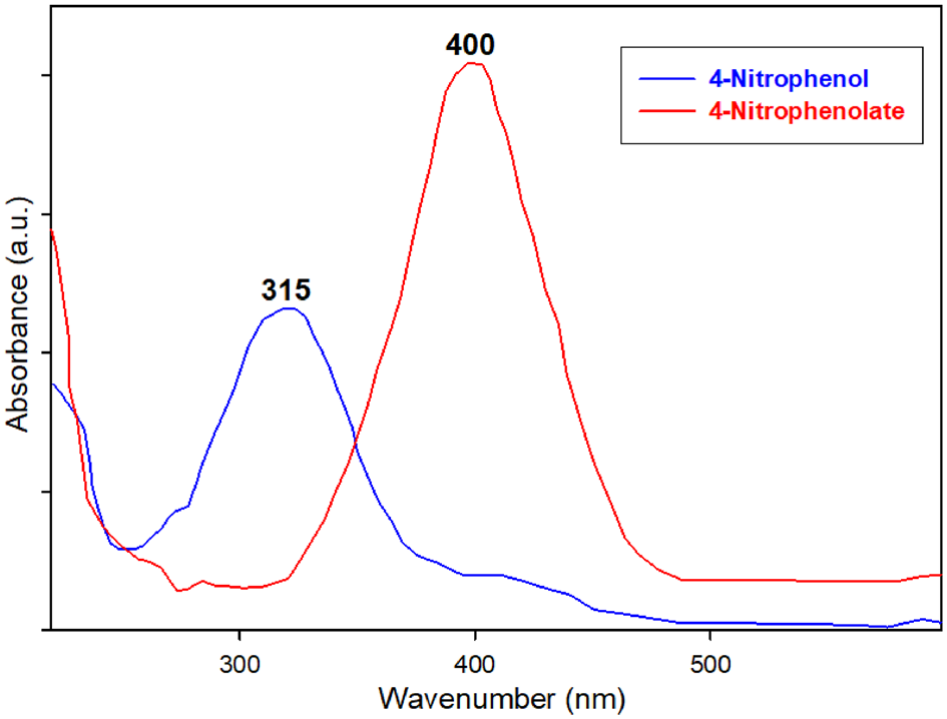
UV–Vis graph of 4-nitrophenol and 4-nitrophenolate.

It is important to note that 4-nitrophenol (4-NP) exhibits bathochromic properties, with its adsorption peak appearing at 315 nm approximately. The addition of NaBH_4_ causes 4-NP to undergo rapid conversion into 4-nitrophenolate (4-NPT), thereby resulting in a shift of the adsorption peak to 400 nm^50^. This conversion is attributed to the ionization of 4-NP into 4-nitrophenolate ion (4-NPT) under alkaline conditions (Fig. 8). Moreover, the initial yellow solution of the 4-NP solution intensifies to a bright yellow upon the formation of 4-NPT. Hence, to track the progress of the catalytic reduction of 4-NP, alterations in the 4-NPT peak at 400 nm were monitored via UV–Vis spectroscopy. Upon completion of the reaction, the peak observed at 400 nm vanished, and in its place, a fresh peak appeared at 300 nm approximately, indicating the successful formation of 4-aminophenol (4-AP). The fast disappearance of the bright yellow color of the solution in the presence of the utilized Cu-Zn-MOFs as catalyst indicates the high catalytic activity of Cu-Zn-MOFs in the degradation of OPDs^50^.

**Figure 9:**
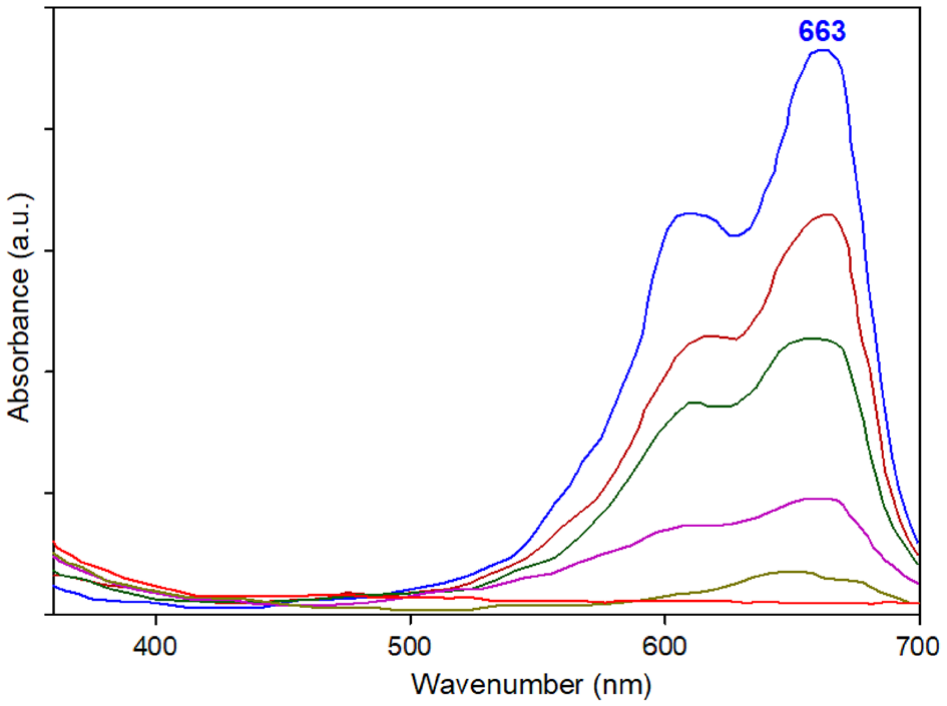
UV–Vis spectrum of MB during the degradation reaction.

In the study of degradation and reduction of other dyes, similar conditions were applied in terms of decreasing absorption levels at their respective wavelengths, and the results were investigated and analyzed. For example, Fig. 9 presents the alterations observed in the ultraviolet spectrum of MB during the catalytic degradation process. Notably, the absorption intensity experiences a gradual decline over the reaction time, indicative of the progressive transformation occurring.

**Figure 10:**
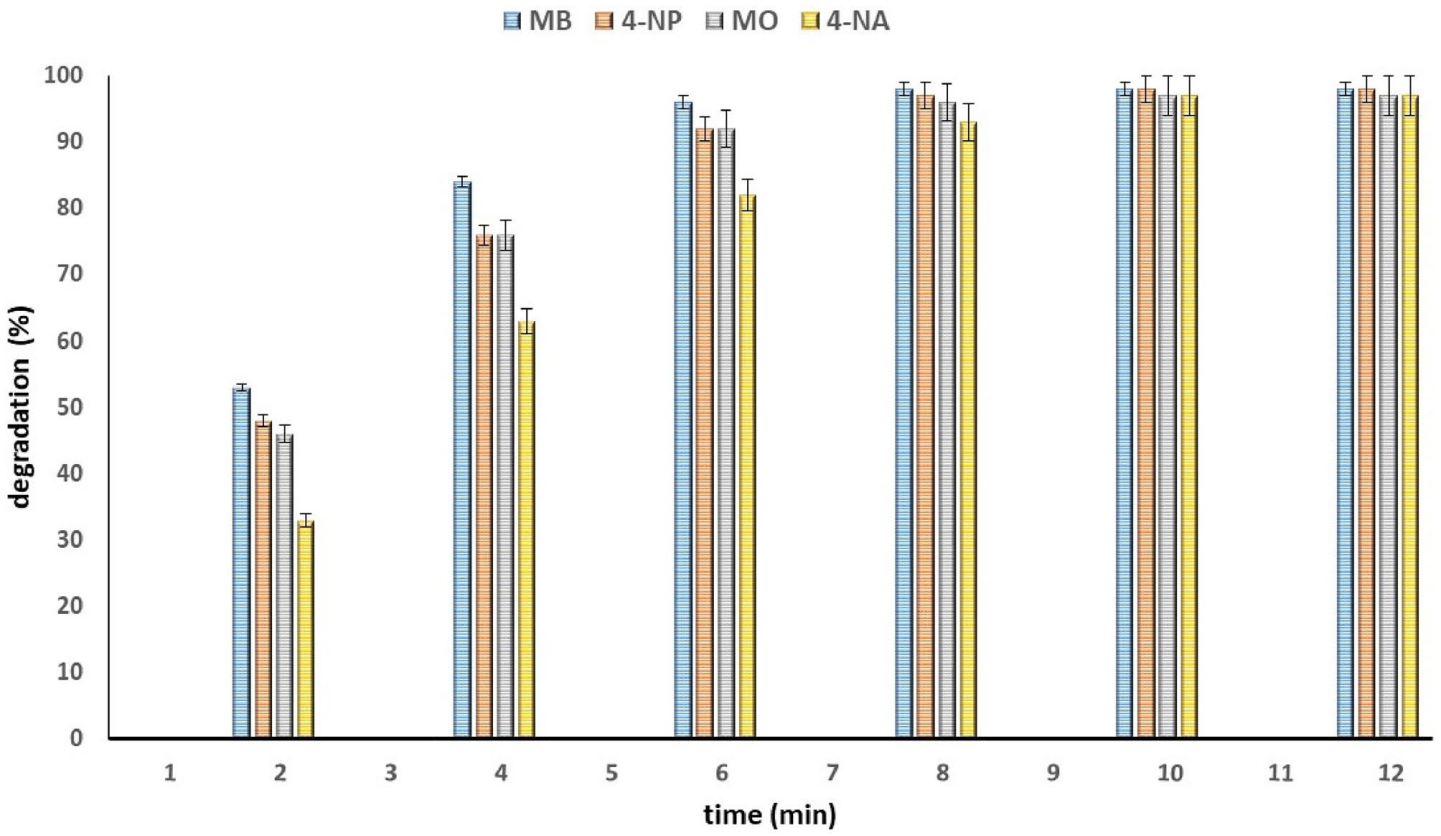
Catalytic degradation reaction of OPDs (MB, MO, 4-NP and 4-NA) in the presence of Cu-Zn-MOF.

During the experimental conditions, the soluble color vanished in 6–12 min, and the concentration of the OPD decreased gradually as the reaction proceeded (Fig. 10).

To analyze the reaction kinetics of the reduction and degradation of the utilized dyes, a graph was plotted by plotting the concentration of the organic dye against reaction time. Due to the significantly higher concentration of NaBH_4_ (0.1 M) compared to that of the dye, the reduction process was considered as a quasi-first-order reaction.

**Figure 11:**
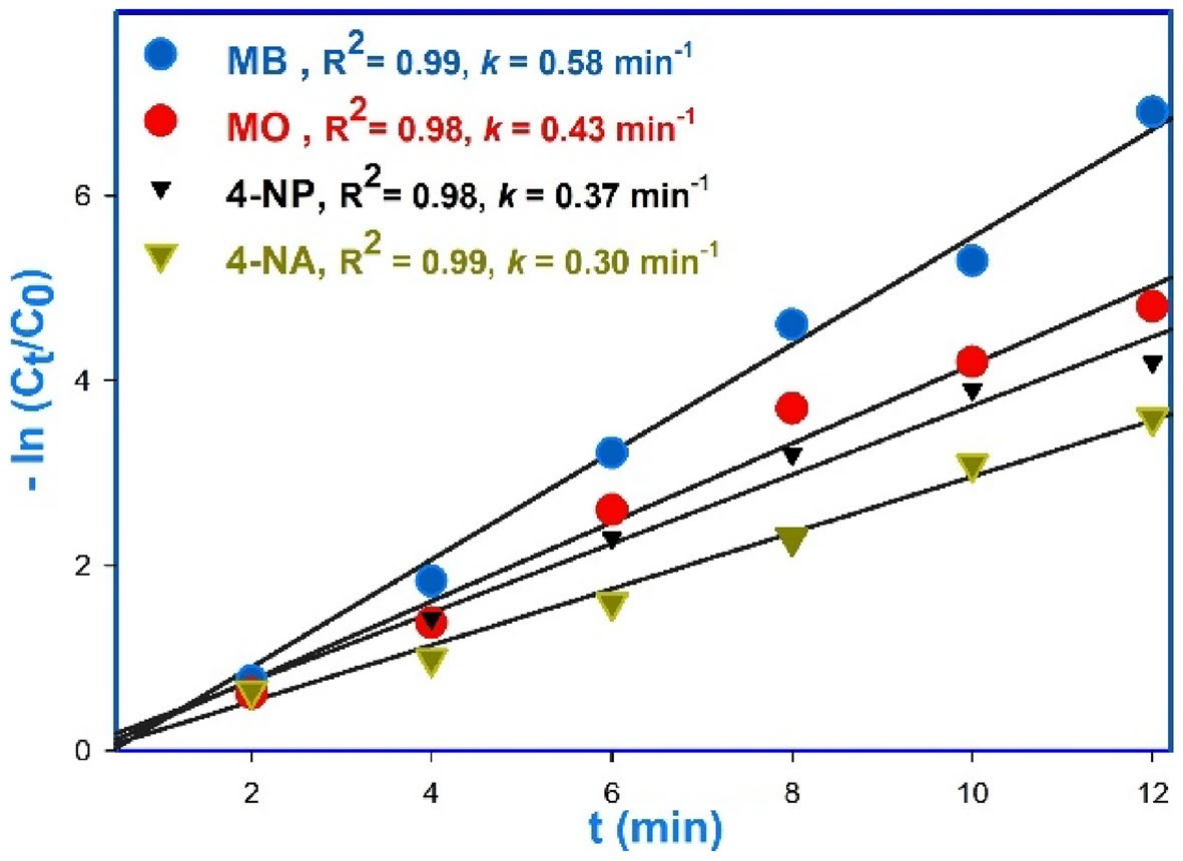
Calculation of degradation reaction rate constant (*k*) using regression diagram.

Using followed equation and the relationship between adsorption intensity and reactant concentration in a quasi-first-order reaction (Fig. 11), the ln ([C_t_]/[C_o_]) graph was plotted against reaction time. The semi-first order kinetic model showed excellent linearity and a strong correlation coefficient (R^2^) close to unity, indicating its effectiveness in representing the complete degradation of OPDs in the dye degradation reaction. Compared to other models, the semi-first order kinetic model outperformed them and provided a comprehensive understanding of the process. In Fig. 8, the measured data were fit to a linear trend line, and the slope of the line was used to obtain the reaction rate constant (*k*, min^−1^)^51^. [A_t_] the initial moment of the reaction, the concentration and intensity of adsorption are represented by [C_o_] and [A_o_], respectively. Also, during the reaction, the same values are represented by [C_t_] and [A_t_].

$$ \ln \;\left( {A_{t} /A_{o} } \right) = \ln \;\left( {\left[ {C_{t} } \right]/\left[ {C_{o} } \right]} \right) = - kt $$

Results indicated that the reduction of OPDs by sodium borohydride follows pseudo-first-order kinetics with rate constants (*k*_*app*_) of 0.5818, 0.4265, 0.3726, and 0.3037 min^−1^ for methylene blue (MB), methyl orange (MO), 4-nitrophenol (4-NP), and 4-nitroaniline (4-NA), respectively, as illustrated in Fig. 11.

**Figure 12:**
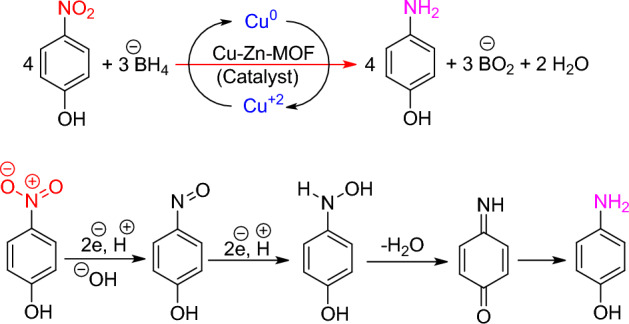
The suggested mechanism for the catalytic reduction of 4-NP.

In the absence of a catalyst, the reduction reaction showed minimal conversion after 5 h. This can be attributed to the significant difference between the levels of the donor (NaBH_4_) and receptor (dye composition). The adsorption intensity at λ_max_ remained almost constant, indicating that the reduction reaction is limited in the absence of a catalyst. In the absence of a catalyst, dye degradation reaction occurs with difficulty in small amounts due to the large distance between the donor (NaBH_4_) and acceptor (dye composition) electron transfer levels^52^. However, the addition of Cu-Zn-MOFs as the catalyst to the reaction mixture reduces the required distance and facilitates electron transfer efficiency. While we have not conducted a thorough investigation into the mechanism of degradation, the literature suggests a possible mechanism for the reduction of 4-NP (as outlined in Fig. 12)^53–55^:

The incorporation of intermediate metal ions, such as Cu^+2^ or Zn^+2^, into the MOF structure enables the interaction with organic dye molecules, resulting in the formation of an active catalytic site. The research findings indicate that the interaction at the copper metal ion site yields a more active catalytic site compared to the interaction with zinc ions. Consequently, this leads to a substantial acceleration in the reaction rate, which can be attributed to the distinct energy level difference between these two metals.

Our research results on reducing organic dyes (MB and 4-NP) using Cu-Zn-MOF catalyst were compared with other reported catalysts and conditions, and Table 3 summarizes the comparison of catalytic conditions. Our investigation has uncovered that Cu-Zn-MOF displays exceptional catalytic performance and achieves faster reaction times in comparison to other catalysts documented in the literature for the degradation of diverse organic dyes.

**Table 3:** Comparison of catalytic reduction of MB and 4-NP in the presence of various catalysts.

OPD	Catalyst (mg)	Conditions	Time (min), k (min^−1^)	Refs.
MB	MPD-Cu^a^ (0.4)	MB (0.1 mL, 200 ppm), NaBH_4_ (0.66 mL, 3 M)	5, 1.44	^56^
	Cu(NPs)/β-CCP^b^ (10)	MB (2 mL, 166 ppm), NaBH_4_ (0.50 mL, 0.04 M)	4, 0.57	^34^
	AgMoOS (10)	MB (100 mL, 20 ppm), NaBH_4_ (2.0 mL, 0.2 M)	6, 0.541	^57^
	NC-AgNPs (50)	MB (2 mL, 20 ppm), NaBH4 (0.95 mL, 0.005 M)	150, 0.16	^58^
	Cu-Zn-MOF (2.8)	MB (20 mL, 20 ppm), NaBH_4_ 23 mL, 0.1 M)	7, 0.58	This work
4-NP	Co/PCNS (0.1)	4-NP (2 mL, 20 ppm), NaBH4 (11 mL, 0.125 M)	7, 0.31	^59^
	AgMoOS (10)	4-NP (100 mL, 20 ppm), NaBH4 (2 mL, 0.2 M)	18, 0.136	^57^
	Cu-NP/C (4.0)	4-NP (1.5 mL, 27.8 ppm), NaBH4 (1.5 mL, 0.02 M)	6, 0.3	^35^
	Cu/MC (0.5)	4-NP (6 mL, 42 ppm), NaBH4 (2 mL, 0.5 M)	5, 0.96	^60^
	Cu-Zn-MOF (2.2)	4-NP (20 mL, 20 ppm), NaBH_4_ 25 mL, 0.1 M)	10, 0.37	This work

^a^Magnetic polydopamine-Cu nanoflowers.

^b^Cu(NPs)/β-Chitin/dicalcium phosphate.

#### Reusability of catalyst

**Figure 13:**
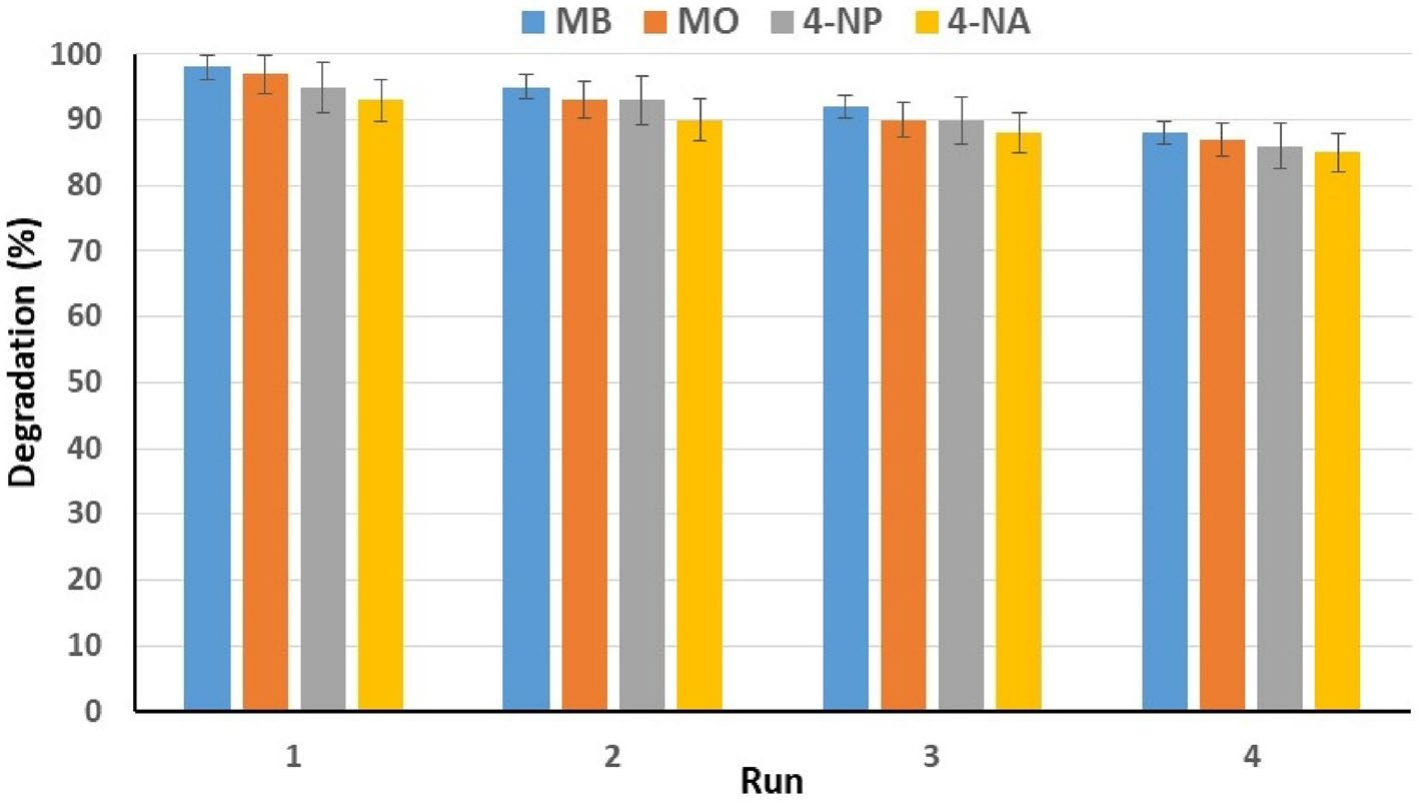
Catalytic efficiency of recycled catalyst.

The long-term and multiple applications of catalysts heavily rely on their stability and reusability. Moreover, the study aimed to evaluate the recycled catalyst's ability to degrade polluting dyes from water. Recycling and reusing the catalyst align with the primary goal of the study, which is to provide a method for eliminating pollutants from water. The use of heterogeneous catalysts can enhance efficiency and cost-effectiveness while reducing environmental impact by minimizing waste and energy consumption. Therefore, we investigated the recyclability of the Cu-Zn-MOF catalyst after degrading dyes with NaBH_4_. Our experiments also demonstrated that MOFs could be recycled for three consecutive runs while maintaining their ability to quantitatively degrade OPDs. Upon the reaction completion, the used catalyst was collected by centrifugation and filtration, washed twice with deionized water and ethanol, dried at 90 °C for 6 h, and then reused for the next run under the same conditions. The catalyst underwent four recovery steps, and its catalytic activity was evaluated. The results showed that the catalyst's efficiency decreased slightly (about 8–10%) after three stages, and the used catalyst (Cu-Zn-MOF) exhibited good results in analyzing important features such as stability, recyclability and reusability (Fig. 13). A possible reason for the decrease in catalyst efficiency is the loss of the catalyst during the recovery and separation process from the reaction mixture.

## Conclusion

In this study, a solvothermal catalytic degradation reaction was used to prepare terephthalic acid from WPT. Copper and zinc salts were used to produce bimetallic MOFs (Cu-Zn-MOFs). The synthesized Cu-Zn-MOF was then utilized as a catalyst to purify wastewater contaminated with OPDs, including MB, MO, 4-NP and 4-NA, through a degradation reaction with sodium borohydride serving as a reducing agent. To demonstrate the potential of MOF-Cu-Zn in wastewater treatment, a simulated solution containing organic dye pollutants, including MB, MO, 4-NP, and 4-NA, was utilized to mimic industrial wastewater from various sectors, such as textiles. A comparative study conducted in this research investigated the qualitative and quantitative efficiency of Cu-Zn-MOF as a catalyst for the reduction and degradation of the examined dyes. The findings demonstrate the exceptional performance of this catalyst, both in terms of kinetics and quantity, achieving a degradation percentage exceeding 95% in less than 12 min. Consequently, MOF nanostructures with a high surface area, optimized through the incorporation of copper as an active metal particle in the catalytic reaction for dye degradation, can be proposed as an effective and efficient catalyst for the degradation of organic dyes in actual wastewater contaminated with these pollutants. The investigation showed that the catalyst can be efficiently recycled and reused with excellent recovery, stability, and performance. Considering its potential for environmental remediation, this catalyst can be recommended for conducting appropriate reactions. The innovative approach presented in this study utilizes plastic waste as a raw material to create a sustainable catalyst, which effectively degrades toxic pollutants in wastewater, indicating great potential for environmental remediation. Further research is required to optimize the process and evaluate the potential of this approach in large-scale applications. This study highlights the importance of utilizing waste materials for sustainable solutions and offers a promising direction for addressing environmental challenges.

## Data Availability

The datasets used and/or analyzed during the current study available from the corresponding author in reasonable request.

## References

[1] Uddin F (2021). Environmental hazard in textile dyeing wastewater from local textile industry. Cellulose.

[2] Baran T, Karaoğlu K, Nasrollahzadeh M (2023). Nano-sized and microporous palladium catalyst supported on modified chitosan/cigarette butt composite for treatment of environmental contaminants. Environ. Res..

[3] Patra S, Swain SK, Swain SK (2022). Nanohybrid Materials for Water Purification.

[4] Akratos CS, Tekerlekopoulou AG, Vayenas DV (2021). Agro-industrial wastewater treatment with decentralized biological treatment methods. Water.

[5] Henze, M., van Loosdrecht, M. C., Ekama, G. A. & Brdjanovic, D. *Biological Wastewater Treatment* (IWA Publishing, 2008).

[6] Jaleh B (2023). Synthesis of bentonite/Ag nanocomposite by laser ablation in air and its application in remediation. Chemosphere.

[7] Abda A (2015). Mutagenicity and genotoxicity of drinking water in Guelma region, Algeria. Environ. Monit. Assess..

[8] Oller I, Malato S, Sánchez-Pérez J (2011). Combination of advanced oxidation processes and biological treatments for wastewater decontamination: A review. Sci. Total Environ..

[9] Hube S (2020). Direct membrane filtration for wastewater treatment and resource recovery: A review. Sci. Total Environ..

[10] Rashid R, Shafiq I, Akhter P, Iqbal MJ, Hussain M (2021). A state-of-the-art review on wastewater treatment techniques: The effectiveness of adsorption method. Environ. Sci. Pollut. Res..

[11] Zhao C (2021). Application of coagulation/flocculation in oily wastewater treatment: A review. Sci. Total Environ..

[12] Teh CY, Budiman PM, Shak KPY, Wu TY (2016). Recent advancement of coagulation–flocculation and its application in wastewater treatment. Ind. Eng. Chem. Res..

[13] Turano E, Curcio S, De Paola MG, Calabrò V, Iorio G (2002). An integrated centrifugation–ultrafiltration system in the treatment of olive mill wastewater. J. Membr. Sci..

[14] Durán A, Monteagudo JM, San Martín I (2018). Operation costs of the solar photo-catalytic degradation of pharmaceuticals in water: A mini-review. Chemosphere.

[15] Xia C (2023). Latest advances in layered covalent organic frameworks for water and wastewater treatment. Chemosphere.

[16] Li X (2023). Application of heterogeneous catalytic ozonation in wastewater treatment: an overview. Catalysts.

[17] Yuan X (2023). Recent advancements and challenges in emerging applications of biochar-based catalysts. Biotechnol. Adv..

[18] Li Y, Yao B, Chen Y, Zhou Y, Duan X (2023). Metal-organic frameworks (MOFs) as efficient catalysts for electro-Fenton (EF) reactions: Current progress and prospects. Chem. Eng. J..

[19] Shaterian HR, Ahmadian HR, Ghashang M, Doostmohammadi R, Yarahmadi H (2008). Ferric hydrogensulfate as effective and recyclable catalyst for mild dithioacetalization of aldehydes and ketones. Phosphorus Sulfur Silicon Relat. Elem..

[20] Yarahmadi H, Shaterian HR (2012). Basic magnetic nanoparticles as efficient catalysts for the preparation of naphthopyrane derivatives. J. Chem. Res..

[21] Ahmad F (2023). Metal-organic frameworks for electrocatalytic water-splitting: Beyond the pyrolysis. Int. J. Hydrog. Energy..

[22] Huang L (2023). Monolithic covalent organic frameworks with hierarchical architecture: Attractive platform for contaminant remediation. Chem. Mater..

[23] Poonia K (2023). Recent advances in Metal Organic Framework (MOF)-based hierarchical composites for water treatment by adsorptional photocatalysis: A review. Environ. Res..

[24] Rao R (2023). Recent advances of metal-organic framework-based and derivative materials in the heterogeneous catalytic removal of volatile organic compounds. J. Colloid Interface Sci..

[25] Naghdi S (2023). Recent advances in application of metal-organic frameworks (MOFs) as adsorbent and catalyst in removal of persistent organic pollutants (POPs). J. Hazard. Mater..

[26] Chen T, Zhao D (2023). Post-synthetic modification of metal-organic framework-based membranes for enhanced molecular separations. Coord. Chem. Rev..

[27] Chung W-T (2023). Recent advances in metal/covalent organic frameworks based materials: Their synthesis, structure design and potential applications for hydrogen production. Coord. Chem. Rev..

[28] Tang J, Wang J (2020). Iron-copper bimetallic metal-organic frameworks for efficient Fenton-like degradation of sulfamethoxazole under mild conditions. Chemosphere.

[29] Wang J-L, Wang C, Lin W (2012). Metal–organic frameworks for light harvesting and photocatalysis. Acs Catal..

[30] Jiao L, Wang Y, Jiang HL, Xu Q (2018). Metal–organic frameworks as platforms for catalytic applications. Adv. Mater..

[31] He J, Zhang Y, Zhang X, Huang Y (2018). Highly efficient Fenton and enzyme-mimetic activities of NH2-MIL-88B (Fe) metal organic framework for methylene blue degradation. Sci. Rep..

[32] Goswami A (2020). 2D MOFs with Ni (II), Cu (II), and Co (II) as efficient oxygen evolution electrocatalysts: rationalization of catalytic performance vs structure of the MOFs and potential of the redox couples. ACS Appl. Mater. Interfaces.

[33] Sun Y, Zhou J, Liu D, Li X, Liang H (2022). Enhanced catalytic performance of Cu-doped MnFe2O4 magnetic ferrites: Tetracycline hydrochloride attacked by superoxide radicals efficiently in a strong alkaline environment. Chemosphere.

[34] Mulongo-Masamba R, El Hazzat M, El Hamidi A, Halim M, Arsalane S (2019). New functional β-chitin/calcium phosphate as promising support of copper nanocatalyst for the reductive degradation of methylene blue. Int. J. Environ. Sci. Technol..

[35] Pasinszki T (2018). Copper nanoparticles grafted on carbon microspheres as novel heterogeneous catalysts and their application for the reduction of nitrophenol and one-pot multicomponent synthesis of hexahydroquinolines. New J. Chem..

[36] Wang Q (2015). Deep eutectic solvents as highly active catalysts for the fast and mild glycolysis of poly(ethylene terephthalate)(PET). Green Chem..

[37] Wang H, Yan R, Li Z, Zhang X, Zhang S (2010). Fe-containing magnetic ionic liquid as an effective catalyst for the glycolysis of poly(ethylene terephthalate). Catal. Comm..

[38] Wang Q (2012). Urea as an efficient and reusable catalyst for the glycolysis of poly(ethylene terephthalate) wastes and the role of hydrogen bond in this process. Green Chem..

[39] Cabrera-Munguia DA (2021). Potential biomedical application of a new MOF based on a derived PET: synthesis and characterization. Bull. Mater. Sci..

[40] Kang MJ, Yu HJ, Jegal J, Kim HS, Cha HG (2020). Depolymerization of PET into terephthalic acid in neutral media catalyzed by the ZSM-5 acidic catalyst. Chem. Eng. J..

[41] Dikio ED, Farah AM (2013). Synthesis, characterization and comparative study of copper and zinc metal organic frameworks. Chem. Sci. Trans..

[42] John S, Mathew B, Koshy EP, George C (2020). Green synthesis of hierarchically porous Cu-and Zn-MOFs by the combined action of hydroxy double salt and surfactant: An ultrafast method. Mater. Today Proc..

[43] Carson CG (2012). Dopant-controlled crystallization in metal-organic frameworks: The role of copper (II) in zinc 1,4-benzenedicarboxylate. J. Phys. Chem. C.

[44] Varmazyari M, Khani Y, Bahadoran F, Shariatinia Z, Soltanali S (2021). Hydrogen production employing Cu (BDC) metal–organic framework support in methanol steam reforming process within monolithic micro-reactors. Int. J. Hydrog. Energy.

[45] Kobayashi Y, Sakuraba T (2008). Silica-coating of metallic copper nanoparticles in aqueous solution. Colloids Surf. A.

[46] Islam MT, Saenz-Arana R, Wang H, Bernal R, Noveron JC (2018). Green synthesis of gold, silver, platinum, and palladium nanoparticles reduced and stabilized by sodium rhodizonate and their catalytic reduction of 4-nitrophenol and methyl orange. New J. Chem..

[47] Sharma K, Singh G, Singh G, Kumara M, Bhalla V (2015). Silver nanoparticles: Facile synthesis and their catalytic application for the degradation of dyes. RSC Adv..

[48] Liu H, Zhong L, Govindaraju S, Yun K (2019). ZnO rod decorated with Ag nanoparticles for enhanced photocatalytic degradation of methylene blue. J. Phys. Chem. Solids.

[49] Melinte V, Stroea L, Buruiana T, Chibac AL (2019). Photocrosslinked hybrid composites with Ag, Au or Au-Ag NPs as visible-light triggered photocatalysts for degradation/reduction of aromatic nitroderivatives. Eur. Polym. J..

[50] Torres Landa SD, Kaur I, Agarwal V (2022). Pithecellobium dulce leaf-derived carbon dots for 4-nitrophenol and Cr (VI) detection. Chemosensors.

[51] Khan MN, Bashir O, Khan TA, Al-Thabaiti SA, Khan Z (2017). Catalytic activity of cobalt nanoparticles for dye and 4-nitro phenol degradation: A kinetic and mechanistic study. Int. J. Chem. Kinet..

[52] Shi Y (2019). Electronic metal–support interaction to modulate MoS2-supported Pd nanoparticles for the degradation of organic dyes. ACS Appl. Nano Mater..

[53] Ahmed Zelekew O, Kuo D-H (2016). A two-oxide nanodiode system made of double-layered p-type Ag_2_O@n-type TiO_2_ for rapid reduction of 4-nitrophenol. Phys. Chem. Chem. Phys..

[54] Shah MT (2017). SiO2 caped Fe3O4 nanostructures as an active heterogeneous catalyst for 4-nitrophenol reduction. Microsyst. Technol..

[55] Kong X, Zhu H, Chen C, Huang G, Chen Q (2017). Insights into the reduction of 4-nitrophenol to 4-aminophenol on catalysts. Chem. Phys. Lett..

[56] Mohammad M, Ahmadpoor F, Shojaosadati SA (2020). Mussel-inspired magnetic nanoflowers as an effective nanozyme and antimicrobial agent for biosensing and catalytic reduction of organic dyes. ACS Omega.

[57] Abdeta AB (2021). A novel AgMoOS bimetallic oxysulfide catalyst for highly efficiency catalytic reduction of organic dyes and chromium (VI). Adv. Powder Technol..

[58] Edison TNJI (2020). Catalytic degradation of organic dyes using green synthesized N-doped carbon supported silver nanoparticles. Fuel.

[59] Hu L (2022). Cobalt with porous carbon architecture: Towards of 4-nitrophenol degradation and reduction. Sep. Purif. Technol..

[60] Xu P (2020). A facile electrostatic droplets assisted synthesis of copper nanoparticles embedded magnetic carbon microspheres for highly effective catalytic reduction of 4-nitrophenol and Rhodamine B. Mater. Chem. Phys..

